# Detection of epithelial to mesenchymal transition in airways of a bleomycin induced pulmonary fibrosis model derived from an α-smooth muscle actin-Cre transgenic mouse

**DOI:** 10.1186/1465-9921-8-1

**Published:** 2007-01-07

**Authors:** Zhuang Wu, Leilei Yang, Lin Cai, Min Zhang, Xuan Cheng, Xiao Yang, Jun Xu

**Affiliations:** 1 Guangzhou Institute of Respiratory Disease, First Affiliated Hospital of Guangzhou Medical College, Guangzhou, 510120, P. R. China; 2 Genetic Laboratory of Development and Diseases, Institute of Biotechnology, 20 Fengtai Eastern Street, Beijing, 100071, P.R.China

## Abstract

**Background:**

Epithelial to mesenchymal transition (EMT) in alveolar epithelial cells (AECs) has been widely observed in patients suffering interstitial pulmonary fibrosis. In vitro studies have also demonstrated that AECs could convert into myofibroblasts following exposure to TGF-β1. In this study, we examined whether EMT occurs in bleomycin (BLM) induced pulmonary fibrosis, and the involvement of bronchial epithelial cells (BECs) in the EMT. Using an α-smooth muscle actin-Cre transgenic mouse (α-SMA-Cre/R26R) strain, we labelled myofibroblasts in vivo. We also performed a phenotypic analysis of human BEC lines during TGF-β1 stimulation in vitro.

**Methods:**

We generated the α-SMA-Cre mouse strain by pronuclear microinjection with a Cre recombinase cDNA driven by the mouse α-smooth muscle actin (α-SMA) promoter. α-SMA-Cre mice were crossed with the Cre-dependent LacZ expressing strain R26R to produce the double transgenic strain α-SMA-Cre/R26R. β-galactosidase (βgal) staining, α-SMA and smooth muscle myosin heavy chains immunostaining were carried out simultaneously to confirm the specificity of expression of the transgenic reporter within smooth muscle cells (SMCs) under physiological conditions. BLM-induced peribronchial fibrosis in α-SMA-Cre/R26R mice was examined by pulmonary βgal staining and α-SMA immunofluorescence staining. To confirm in vivo observations of BECs undergoing EMT, we stimulated human BEC line 16HBE with TGF-β1 and examined the localization of the myofibroblast markers α-SMA and F-actin, and the epithelial marker E-cadherin by immunofluorescence.

**Results:**

βgal staining in organs of healthy α-SMA-Cre/R26R mice corresponded with the distribution of SMCs, as confirmed by α-SMA and SM-MHC immunostaining. BLM-treated mice showed significantly enhanced βgal staining in subepithelial areas in bronchi, terminal bronchioles and walls of pulmonary vessels. Some AECs in certain peribronchial areas or even a small subset of BECs were also positively stained, as confirmed by α-SMA immunostaining. In vitro, addition of TGF-β1 to 16HBE cells could also stimulate the expression of α-SMA and F-actin, while E-cadherin was decreased, consistent with an EMT.

**Conclusion:**

We observed airway EMT in BLM-induced peribronchial fibrosis mice. BECs, like AECs, have the capacity to undergo EMT and to contribute to mesenchymal expansion in pulmonary fibrosis.

## Background

Myofibroblast cells, an intermediate cell type between fibroblasts and smooth muscle cells (SMCs), have been suggested to play an important role in the development of interstitial pulmonary fibrosis (IPF), which produces excessive amounts of extracellular matrix (ECM), leading to formation of fibroblastic foci [1-3]. However, much is still unknown regarding the origin of myofibroblasts and the process resulting in devastating airway aggravation. Previously, it was suggested that peribronchiolar and perivascular fibroblasts transdifferentiate into myofibroblasts following exposure to profibrotic mediators such as TGF-β1 [4]. Alternatively, airway SMCs might dedifferentiate into myofibroblasts, but this possibility has been ruled out by several studies suggesting that ultrastructural features and ECM expression profiles of myofibroblasts are more similar to fibroblasts than to SMCs [1,5]. Recently, fibrocytes originating in the bone marrow have been proposed to be recruited into the lung after bleomycin (BLM) administration and to act as myofibroblast progenitors [6]. More recently, alveolar epithelial cells (AECs) have been shown to undergo epithelial to mesenchymal transition (EMT) to produce myofibroblasts in IPF patients and following TGF-β1 treatment in vitro [7-9]. Moreover, EMT in AECs has been demonstrated in a mouse pulmonary fibrosis model [10]. The BLM induced peribronchial fibrosis mouse model largely recapitulates histological features of human pulmonary fibrosis [11], and thus provides a convenient and powerful in vivo tool that has been the most widely used animal model to study the pathogenetic mechanisms of pulmonary fibrosis. However, the common BLM-induced pulmonary fibrotic model is derived from wild mouse and thus is unsuitable for tracking the origin of active myofibroblasts in the development of pulmonary fibrosis, due to their great "plasticity" and tendency to switch to other phenotypes [12].

In the present study, we employed the Cre/LoxP recombinase system, using the α-smooth muscle actin (α-SMA) promoter to drive Cre-dependent recombination in presumptive myofibroblast cells as well as SMCs. We then generated an α-SMA-Cre/R26R transgenic mouse strain that allows permanent β-galactosidase (βgal) labeling in airway SMCs and the other structural cells undergoing transdifferentiation into myofibroblasts. Since the recombination is achieved by Cre-dependent removal of the transcriptional stop sequence between the two LoxP sites upstream of the lacZ gene in R26R mice, lacZ expression will permanently label Cre-expressing cells [13,14]. As expected, our transgenic mouse model accurately labeled the distribution of SMCs in various organs under physiological conditions; cumulatively recorded the activation of myofibroblasts in the lung under BLM induced fibrotic conditions and revealed EMT occurring in AECs and even in BECs. Moreover, to verify the occurrence of EMT in BECs in vitro, we treated the human BEC cell line 16HBE with TGF-β1, which was also capable of inducing EMT.

## Methods

### Reagents

For histological immunofluorescent staining, anti-α-SMA monoclonal antibody (mAb) was purchased from Sigma (reactive with human and mouse α-SMA, Cat A2547); anti-bovine smooth muscle myosin heavy chains (SM-MHC) polyclonal antibody (pAb) was kindly provided by Professor Mary Anne (NIH/NHLBI, US); rabbit anti-human E-cadherin pAb was purchased from Santa Cruz Biotechnology (Cat sc-7870), rabbit anti-mouse/human E-cadherin pAb was purchased from Boster Company (Cat BA0475). GAPDH mAb was purchased from Chemicon (Cat CB1001). Secondary antibodies of goat anti-rabbit pAb conjugated with FITC and goat anti-mouse pAb conjugated with TRITC were purchased from Bethyl(Cat A120-201F) and Open Biosystems (Cat SAB1428), respectively. Goat anti-mouse pAb conjugated to HRP was purchased from Santa Cruz Biotechnology (Cat sc-2005). Bleomycin (BLM) used for establishing the peribronchial fibrosis model was purchased from Nipponkayaku (Tokyo, Japan). Primers were synthesized in Sangon (Shanghai, China). All chemicals for βgal staining were purchased from Jingmei Company (Shenzhen, China).

### Generation of the α˜
 MathType@MTEF@5@5@+=feaafiart1ev1aaatCvAUfKttLearuWrP9MDH5MBPbIqV92AaeXatLxBI9gBaebbnrfifHhDYfgasaacH8akY=wiFfYdH8Gipec8Eeeu0xXdbba9frFj0=OqFfea0dXdd9vqai=hGuQ8kuc9pgc9s8qqaq=dirpe0xb9q8qiLsFr0=vr0=vr0dc8meaabaqaciaacaGaaeqabaqabeGadaaakeaacuaHXoqygaacaaaa@2E5A@-SMA-Cre/R26R transgenic mouse strain

**Figure 1:**
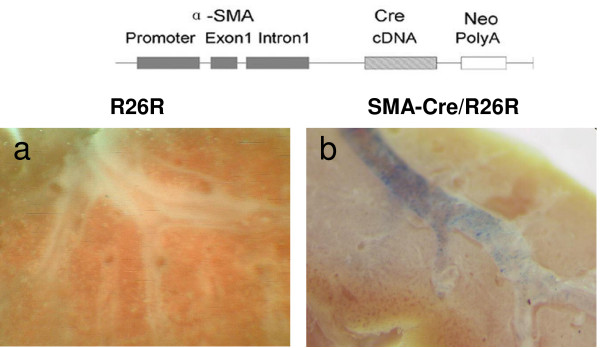
**Transgene construction and βgal staining of lung lobes**. A. Transgene fragment for microinjection. The Cre cDNA and a Neo polyadenylylation signal were placed under the control of the mouse α-SMA promoter (-1070 to +2582, including the first exon and part of the first intron). B. Comparison of βgal staining in the bronchi of R26R and α-SMA-Cre/R26R mice. Positive βgal staining (blue color) is observed in the bronchi of α-SMA-Cre/R26R mice (a, 20× magnification), but not in R26R mice (b, 20× magnification).

The Cre recombinase cDNA was PCR amplified from the pMCI-13Cre plasmid (a gift from Professor F. Costantini, Department of Genetics, Columbia University, NY, USA) using the following primers: forward 5'-GAAGATCTATGCCCAAGAAGAAGAGGAAGGTGTCCAATTTACTGAC-3' and reverse 5'-CGGAATTCTGAACAAACGACCCAAC-3'. The PCR product was then sub-cloned into the BamHI-EcoRI site of the VSMP8 plasmid (a gift of Professor Art Strauch, Dorothy M. Davis Heart and Lung Research Institute, Columbus, OH, USA) which contains the mouse αSMA promoter fragment -1070~+2582, including the first exon and part of first intron (GenBank: U63129 and M57409). The α-SMA promoter-Cre fragment was released from the construct using Sphl and EcoRI for transgenic microinjection (Fig. 1). Transgenic α-SMA-Cre mice were produced by pronuclear injection of the recombinant DNA fragment into fertilized F2 eggs of CBA mice using standard microinjection techniques. Offspring from an α-SMA-Cre-carrying transgenic founder mouse were selected and crossed to the Cre dependent conditional reporter strain R26R+/+ (Rosa26, Soriano P)[15].

### Generation of the BLM-induced pulmonary fibrosis mouse model

5–6 wk old SMA-Cre/R26R mice were endotracheally injected with 80 μl BLM (3 mg/kg in PBS) or with 80 μl PBS (n = 4 for each group). These mice were sacrificed 20 days later for western blot analysis, βgal and immunofluorescent staining.

### Tissue β-galactosidase (βgal) staining

Organs were dissected from BLM or PBS treated transgenic mice and subjected to βgal staining. Briefly, organs were fixed in 0.1 M PBS, pH7.3 containing 0.25% glutaraldehyde, 2 mM MgCl2, 5 mM EGTA at 4°C for 1–2 hrs. Left lung lobes were perfused with 1 ml fixing solution by endotracheal injection and right lobes were ligated and removed. Tissues were then incubated in wash buffer (0.1 M PBS, pH7.3 with 2 mM MgCl2, 0.01% deoxycholate, 0.02%NP-40) 3 times for 30 min each, and then in staining buffer (0.1 M PBS, pH7.3 with 1 mg/ml βgal, 2 mM MgCl2, 0.01% deoxycholate, 5 mM K3Fe(CN)6, 6 mM K4Fe(CN)6, 0.02% NP-40) at 37°C overnight. Following staining, wholemount tissues were observed under XTL-3400 Zoom Stereo Microscope (CANY, Shanghai, China) or processed by dehydrating, wax embedding, sectioning at 8 μm intervals and counterstaining with Carmine Alum. Microscopic analyses were performed with a Leica DM LB2 microscope equipped with a digital camera.

### Lung histology and immunohistochemistry

After sacrificing α-SMA-Cre/R26R mice, right lung lobes (upper and middle lobes) were dissected and fixed in formalin and processed by conventional histological procedures. After sectioning at 4 μm intervals, sections were dewaxed, rehydrated, blocked with 10% goat serum for 60 min at room temperature and immunofluorescently stained with α-SMA, SM-MHC or E-cadherin. Sections were incubated with anti-α-SMA mAb (1:400), anti-bovine SM-MHC pAb (1:400) or co-incubated with E-cadherin pAb (1:100) overnight at 4°C and subsequently incubated with goat anti-mouse IgG-TRITC (1:800) pAb or goat anti-rabbit IgG-FITC (1:400) pAb for 1 hour. DAPI was used to stain nuclei (500 ng/ml in 95% ethanol) for 20 sec, and coverslips were mounted with 80% glycerol. Slides were examined using a Leica DC 500-fluorescence microscope equipped with a digital camera.

Alternatively, lung sections were processed for Masson's trichrome staining to detect collagen and elastin. The staining was carried out using Masson trichrome staining Kit (Maxim-Bio, Fuzhou, China) according to the manufacturer's instruction.

### Western Blot

α-SMA protein levels in lungs were evaluated by western blot as previously described [16]. After cytoplasmic protein extraction from the lower lobe of right lung of PBS or BLM-injected mice, protein was quantified using a BCA assay kit (Pierce, USA) and 20 μg was used for SDS-PAGE electrophoresis. Following electrophoretic transfer, membranes were incubated with anti-α-SMA mAb (1:1000) in TBS/T buffer at 4°C overnight. Membranes were incubated with anti-mouse IgG secondary antibody conjugated to HRP (1:1000), followed by exposure to ECL chemiluminescent substrate (Amersham, UK) and digital scanning in Image station 2000 (Kodak, US). Following α-SMA blotting, films were placed in stripping buffer (50 mM DTT, 50 mM Tris. HCI,2%SDS) at 50°C for 30 minutes, washed 5 times, reblocked and reprobed with GAPDH mAb (1:800) and HRP conjugated secondary antibody. Then the membranes went through chemiluminescence as discribed above to detect GAPDH protein in the same film. α-SMA protein levels were measured by densitometry, and expressed relative to GAPDH. Duplicate samples were analyzed for each mouse.

### Cell culture and immunofluorescent staining

The human bronchial epithelial cell line 16HBE-14o (16HBE), a generous gift from Professor S. Holgate (Southampton University, UK) was routinely maintained in growth medium consisting of MEM (Life Technologies, USA) and 10% FCS (Shijiqing Co, China). Cells were seeded into sterile round coverslips placed inside 12-well plates. On reaching 70% confluence, medium was changed to FCS-free MEM, and rhTGF-β1 (R&D company, US) was added to a subset of wells to a final concentration of 10 μg/L. 72 hours later, all wells were washed twice with cold PBS and a subset of wells were fixed with cold methanol:acetone (1:1) at -20°C for 10 min. Coverslips were removed from the wells and placed on glass slides, blocked with 10% goat serum for 60 min. Cells on coverslips were incubated with anti α-SMA mAb (1:400) or rabbit anti human E-cadherin (1:50) overnight at 4°C and subsequently incubated with goat anti-mouse IgG-TRITC (1:800) or goat anti-rabbit IgG-FITC (1:400) for 1 hour. Another subset of wells were fixed with PFA at RT for 20 min and treated with 0.1% TritonX-100 for 5 min. Coverslips were removed from wells, placed on slides, blocked with 10% goat serum for 30 min and incubated with 100 μl Alex 594 phalloidin (1:500) for 20 min at RT. DAPI was used to stain nuclei (500 ng/ml in 95% ethanol) for 20 sec, and coverslips were mounted with 80% glycerol. Slides were examined using a Leica DC 500-fluorescence microscope equipped with a digital camera.

## Results

### Generation of α-SMA-Cre/R26R transgenic mice

To permanently label myofibroblasts, we firstly generated transgenic mice bearing an α-SMA promoter driven Cre. Ten pseudopregnant mice were implanted oviductally with fertilized eggs injected with the construct, yielding 19 offspring, 4 of which were identified to carry the randomly integrated transgene. Two founder mice were selected and used to produce inbred strains. We then crossed an α-SMA-Cre transgenic strain to reporter strain R26R+/+ whereby Cre-specific recombination at the ROSA26 locus allows expression of β-galactosidase in smooth muscle cells and myofibroblasts.

### βgal staining corresponds with distribution of the smooth muscles of the α-SMA-Cre R26R strain

βgal staining was performed on offspring of the α-SMA-Cre/R26R and the R26R mice respectively. Positive βgal staining was observed in the trachea of the α-SMA-Cre/R26R strain (Fig. 1b), but not in that of the R26R mouse (Fig. 1a) under anatomy microscopy, confirming that the βgal staining resulted from α-SMA-driven Cre-mediated recombination.

**Figure 2:**
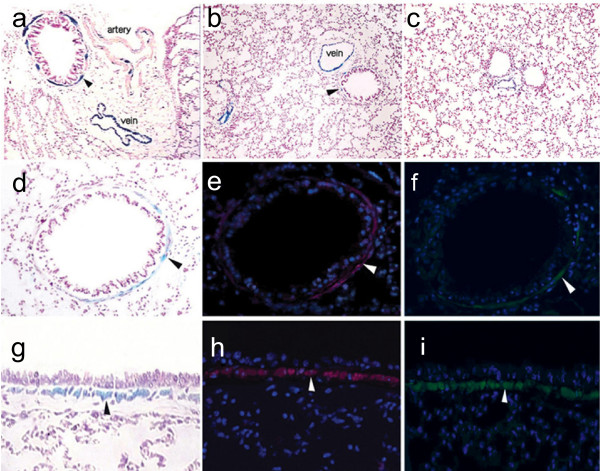
**βgal and immunofluorescent staining in lung tissues of α-SMA-Cre/R26R mice**. In βgal stained sections (a, b, c, d, g), intrapulmonary veins were homogeneously stained (a, b) and pulmonary arteries were heterogeneously stained (a). In the main bronchus of pulmonary hilum, unstained ciliated epithelia were surrounded by a βgal stained muscular layer (a, arrowhead), βgal staining was not detected in terminal bronchioles, although small veins were positively stained (c). The thin layer of βgal staining was observed in the sub-epithelial areas of small and medium bronchi, respectively (arrowheads in b, d, g). The βgal stained areas of bronchus (d, g) paralleled the staining pattern for α-SMA (TRITC-labeled, arrowheads in e, h) and SM-MHC (FITC-labeled, arrowheads in f, i) (a-c, 100×, d-i, 400× magnification).

As expected, βgal staining was highly restricted to SMCs in smooth muscle-rich organs isolated from the α-SMA-Cre/R26R mice (data not shown). In pulmonary arteries and veins, βgal staining was consistent with the natural distribution of smooth muscle tissue at these sites (Fig. 2a, b). In the intrapulmonary bronchus (Fig. 2d, g), the staining was precisely localized to the muscularis layer of the airway, which paralleled the immunofluorescent staining pattern of α-SMA (Fig. 2e, h) and SM-MHC (Fig. 2f, i). Neither the terminal bronchioles, which lack SMC (Fig. 2c), nor the bronchial epithelia (Fig. 2a,b,c,d,g) showed positive βgal staining.

### A great enhancement of βgal staining in the lungs of the double transgenic mice after Bleomycin treatment

**Figure 3:**
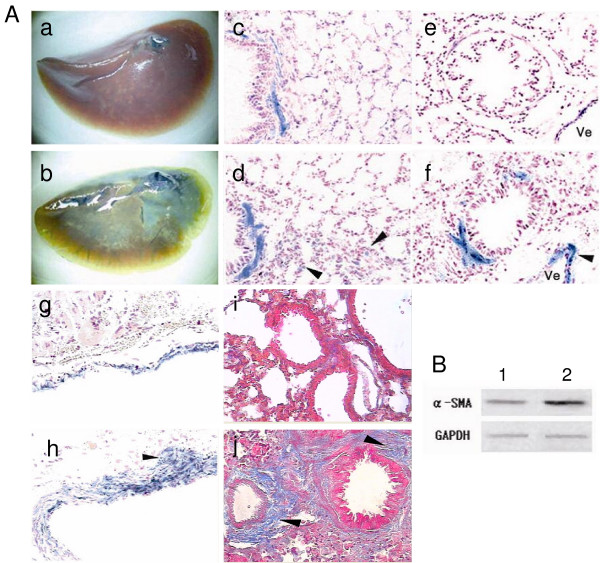
**Effects of BLM on lung α-SMA protein levels, ECM deposition and βgal staining**. Panel A: βgal and Masson's trichrome-staining in sections of lung tissue shows βgal staining to wholemount left lung lobes of the PBS-treated (a) and the BLM-treated mice (b) (a, b 10× magnification). In moderate bronchi, thickened bronchial wall with homogeneous βgal stained fusiform cells was observed in the BLM-treated lungs (d), and not in the PBS-treated mouse (c), Arrowheads indicate that a few cells in alveolar wall were positively stained (d). In pulmonary bronchioles and vessels, BLM treated lung demonstrated enhanced βgal expression (f, h), compared with that of PBS treated lung (e, g). βgal stained venous wall was thickened (arrowhead in f) and the positively stained cells infiltrated outwards (arrowhead in h). In the Masson's trichrome-stained lung sections (i, j), extensive collagen staining (Blue color) was seen in BLM treated lung (arrowheads in j), but not in the control with PBS (i). (c-j 400× magnification). Panel B: Western blot analysis of protein extracts from lower right lobes of the BLM treated mice (2) and control mice (1).

The double transgenic mouse strain described above provides a simple means to follow expression of α-SMA, and thus the regulation of myofibroblast development during pulmonary fibrosis. We next endotracheally administrated BLM in the transgenic mice for induction of lung injury and fibrosis. As shown in figure 3A~b, in the wholemount lung preparations from BLM-treated α-SMA-Cre/R26R transgenic mice, βgal staining is easily observed. In contrast, except for the helium area, the staining is not observed in preparations from PBS-treated transgenic mice (Fig. 3A~a).

Histological observations reveal a significantly increased number of βgal staining- positive cells located at subepithelial areas of bronchioles, terminal bronchioles (Fig. 3A~d,f) and tunica media of pulmonary vessels, particularly pulmonary veins (Fig. 3A~f, h arrowheads) in BLM treated mice. At these sites, distribution of collagen is visualized by Masson's trichrome staining, showing increased collagen deposition around the walls of small veins and terminal respiratory bronchioles and in certain parenchymal areas (Fig. 3A~j arrowheads). In contrast, there is only minimal βgal staining (Fig. 3A~c, e, g) and Masson trichrome staining (Fig. 3A~i) in the lung of control mice.

Correspondingly, western blot analysis revealed an overall increase in lung α-SMA protein in the BLM treated transgenic mice, compared with the control mice (Fig. 3B)

### Detection of EMT in bronchial epithelial cells of the α-SMA-Cre/R26R mice during BLM-induced pulmonary fibrosis

**Figure 4:**
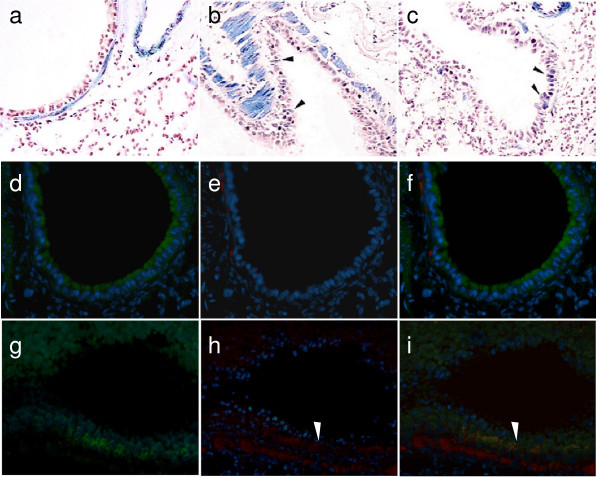
**βgal and αSMA positively stained bronchial epithelial cells in the α-SMA-Cre R26R mice treated with BLM**. βgal stained lung sections of α-SMA-Cre/R26R mice without and with BLM treatment (a-c). The section from BLM treated mice showed a few βgal stained bronchial epithelial cells (arrowheads in b and c), but not from PBS treated mice (a). Double immunofluorescent staining for α-SMA and E-Cadherin was performed on the sections from PBS (d-f) or BLM (g-i) treated mice. d, g: FITC-labeled E-cadherin; e, h: TRITC labeled α-SMA. Positive double immunofluorescent staining (g, h, i) was observed in the bronchial epithelial cells lining the bronchioles of the BLM-treated lung where βgal staining was detected as above, but not in the control (d, e, f). The images of (d) and (e), or (g) and (h) were merged into (f) and (i). The red fluorescence (h arrowhead) indicated the positive α-SMA staining and yellow fluorescent staining (i arrowhead) indicated that the epithelial cells positively co-stained with α-SMA and E-Cadherin. (all images are 400× magnification).

As shown in Fig. 4, we also detected a few βgal positive cells, with basal or columnar epithelial cell morphology, existing in epithelia lining the bronchioles (Fig. 4b, c arrowheads) and air-sacs (Fig. 3A~d arrowheads), in the BLM-treated transgenic mice. This was not observed in the control mice (Fig. 3A~c,e; Fig. 4a). Using double immunofluorescent staining, with antibodies against α-SMA and E-cadherin, we further demonstrated that certain cells located at bronchiolar epithelium of BLM treated mice were simultaneously stained with these epithelial and mesenchymal markers (Fig. 4g,h,i arrowheads), indicating their undergoing of EMT. No common-staining was found in control mice (Fig. 4d,e,f). We have also found similar results from lung tissues after BLM treatment at day 7, 14, 20 and 28.

### In vitro phenotype analysis of 16HBE following exposure of TGF-β1

**Figure 5:**
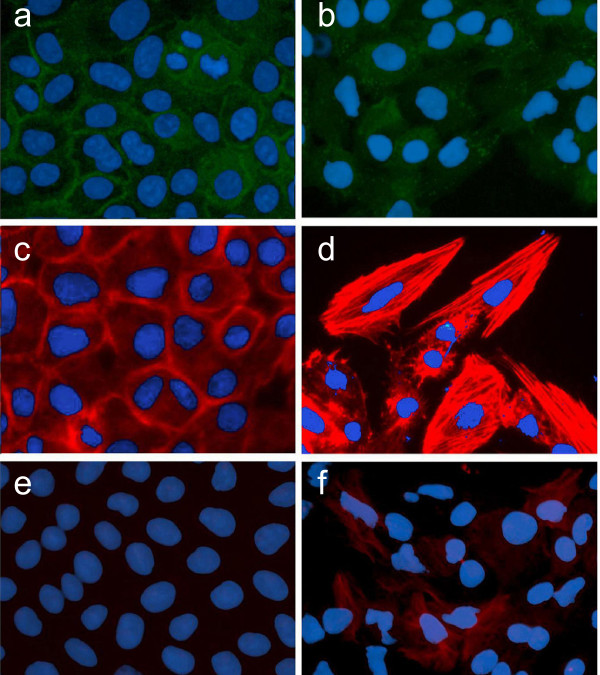
**Phenotypic analysis of the human bronchial epithelial cell line (16HBE) following exposure to TGF-β1**. Immunofluorescent staining for E-cadherin (a, b) showed that exposure to TGF-β1 (b) resulted in an apparent reduction and redistribution of E-cadherin from intercellular junction areas into cytoplasm, compared to control (a). Mesenchymal marker F-actin, was faintly stained at the cell margin in the control (c), whereas the staining was substantially enhanced and abundantly located throughout cytoplasm after TGF-β1 stimulation (d). Immunofluorescent staining for (-SMA was not detected in the cells under basal conditions (e), but was observable in a few cells after TGF-β1 exposure (f).

In vitro immunofluorescent staining of 16HBE cells (human bronchial epithelial cell line) demonstrates that exposure to TGF-β1 results in an apparent reduction of E-cadherin staining, an epithelial marker, concomitant with its redistribution from intercellular junction areas into the cytoplasm (Fig. 5a, b). In contrast, the mesenchymal marker F-actin, whose expression was detectable only at the cellular margin before the exposure, shows an increased level in the epithelial cells where it is diffusely distributed throughout the cytoplasm after stimulation with TGF-β1 (Fig. 5c, d). Meanwhile, positive α-SMA immunofluorescent staining, which was undetectable prior exposure to TGF-β1, appeared in the cytoplasm in a small number of the 16HBE cells (Fig. 5e, f).

## Discussion

Using the Cre/Loxp system, we generated a transgenic mouse strain that expressed lacZ specifically in SMCs and myofibroblasts containing tissues. The βgal expression pattern in the α-SMA-Cre/R26R transgenic model closely resembled the expression of endogenous α-SMA in the airways, and that in the gastrointestinal channel, vessels and genitourinary tract under normal physiological conditions. These data suggest that the SMP8 promoter region of the α-SMA gene, including the first exon and part of the first intron (-1070 to +2582 of the mouse α-SMA promoter), is sufficient to recapitulate endogenous α-SMA expression patterns, in concordance with previous studies [17-19].

Smooth muscle-targeted Cre recombinase mice that have previously been generated by others for study of diseases, including SM22-CreER and SMMHC-Cre strains in which Cre is driven by the promoter of SM22 gene or SM-MHC gene, respectively. Feil and colleagues have generated the SM22-CreER transgenic mice to the effect that the expression of the transgene is confined to smooth muscle cells for studying vascular and gastrointestinal diseases [20]. However, gene knockout studies suggest that SM22 is not required for vascular and visceral SMC homeostatic functions in the developing mouse [21], and there are no data demonstrating that SM22 expression signifies myofibroblast activation. With regards to the SMMHC-Cre strain that has also been used to study vascular development and diseases [22], it has been documented that SM-MHC is seldom expressed in non-SMC cells such as myofibroblasts [23,24]. In contrast, our α-SMA-Cre/R26R strain appears to be sensitive to myofibroblast activation after BLM exposure, as Cre-mediated recombination is controlled by the promoter of the gene encoding α-SMA, a marker of myofibroblast transition.

Additionally, for the reason that in vivo recombination in Cre/Loxp system is irreversible, βgal staining in the lung of our transgenic strain could reflect past and present myofibroblast transition events post BLM treatment. This may assist discovery of the cellular source of the active myofibroblasts in the development of pulmonary fibrosis. In the chronic progression of fibrosis, multiple cycles of injury and repair may occur repeatedly with a broad time period and range of sites. Activation of the α-SMA promoter may be a transient event and limited to a subgroup of cells at a given time point [25]. So the α-SMA-Cre mouse strain is likely to be highly relevant for studies of fibrotic diseases and activation of myofibroblasts, and trace the source of myofibroblasts.

In the BLM-treated α-SMA-Cre/R26R mice, we observed a number of βgal staining positive cells emerging in the subepithelial areas of bronchioles and terminal bronchioles and in the ectoblast of vessels, concomitant with extensive Masson Trichrome-stained extracellular matrix. In comparison, in the PBS-treated α-SMA-Cre/R26R mice, βgal positive cells were seldom seen, suggesting that not only can the reporter mice demonstrate the inherent distribution of pulmonary SMCs under physiologic condition, but also have the capability to sensitively record the trail of myofibroblast transition in the lung of the mice following pathologic stimulation. We demonstrate herein that increased βgal expression in the lungs of the BLM-treated mice is mainly to be due to the appearance of myofibroblasts in the subepithelial areas of bronchiole and terminal bronchiole. Previous studies had shown that airway BLM administration does not result in remarkable morphological changes in the SMC layer [26].

There have been prior suggestions that EMT occurs in the lung during fibrogenesis, but these suggestions derive largely from studies of transformed cells or primary AECs cultured on plastic, the in vivo significance of which is unclear [7,9]. It has recently been reported from IPF lung biopsies that epithelial cells had acquired mesenchymal features, raising the possibility of EMT during fibrogenesis [8]. More recently, Kim and colleagues developed a transgenic mouse reporter strain in which lung epithelial cells were genetically altered to permanently express βgal, and their fates are followed in an established model of pulmonary fibrosis induced by intranasal Adeno-TGF-β1. They showed that βgal-positive cells expressing mesenchymal markers accumulated within 3 weeks of in vivo TGF-β1 expression, demonstrating that EMT occurs in vivo in an animal model [10]. As shown at figure 3, we also observed the occurrence of EMT in parenchymal alveloar areas following BLM stimulation in our α-SMA-Cre/R26R reporter mice where a few βgal-positive cells located in alveolar wall demonstrated that the cells were undergoing EMT. Alternatively, the βgal-stained epithelial cells may simply demonstrate transcriptional activation of α-SMA gene in these cells.

Additionally, βgal was stained positively in a few basal epithelial cells and columnar epithelial cells lining the bronchiole during bleomycin-induced lung fibrosis in the reporter mice. The immunofluorescent co-staining of the both E-cadherin and α-SMA confirmed further that the BECs were undergoing EMT. When we focused on the BEC cell line 16HBE in vitro, we found that exposure to TGF-β1 led to a remarkable myofibroblast cell-like phenotype, marked by expression of α-SMA and F-actin and the reduction of the epithelial-specific junction localization of E-cadherin. Taken together, these observations suggest that BECs might also be capable of undergoing EMT and thereby provide another cellular source for the parenchymal aggregation of myofibroblasts during fibrosis.

As mentioned above, however, the present histological observations in the reporter mice do not support the rationale that EMT exerts a critical influence on the progression of pulmonary fibrosis, because EMT indicated by βgal staining and α-SMA immunostaining was rarely detected in BECs and AECs of BLM-treated mice. For the predominant activation of sub-epithelial myofibroblasts in the development of BLM-treated lung fibrosis, fibroblasts or other sources of progenitors may play more essential roles which contribute to the pool of expanded myofibroblasts after lung injury. To what extent does EMT contribute to the aggravation of fibrosis, whether similar EMT in BECs occur in IPF patients are all interesting questions for future studies.

Additionally, the α-SMA-Cre single transgenic strain bearing the α-SMA driven Cre is sufficiently sensitive to test the function of a candidate gene in SMCs or myofibroblasts on the development of pulmonary fibrosis. Tissue-specific gene knockout or knock-in can be accomplished via crossing the α-SMA-Cre mouse to a strain containing a loxP site flanked sequence of interest.

## Conclusion

In conclusion, we have developed a double transgenic reporter mouse strain to map the natural distribution of α-SMA-expressing cells in vivo under basal physiological condition. Moreover, lung cells that do not express α-SMA under normal conditions may permanently express βgal via α-SMA activation in response to pathologic stimulation, thus allowing tracking of the cellular source of myofibroblasts and to definitively test whether EMT occurs in vivo.

## Competing interests

The author(s) declare that they have no competing interests.

## Authors' contributions

ZW carried out the transgene construction, transgenic screening and breeding, histological works and drafted the manuscript. LLY carried out the microinjections and transgenic screening and breeding. LC participated in the histological work and mice screening and breeding. MZ participated in the in vitro immunostaining. XC participated in the microinjections. XY guided the microinjection and animal breeding. JX design the study, technical support the research, revise the manuscript and give final approval of the version to be published. All authors read and approved the final manuscript.
